# Utilizing machine learning techniques to identify severe sleep disturbances in Chinese adolescents: an analysis of lifestyle, physical activity, and psychological factors

**DOI:** 10.3389/fpsyt.2024.1447281

**Published:** 2024-11-07

**Authors:** Lirong Zhang, Shaocong Zhao, Wei Yang, Zhongbing Yang, Zhi’an Wu, Hua Zheng, Mingxing Lei

**Affiliations:** ^1^ Department of Physical Education, Xiamen University of Technology, Xiamen, Fujian, China; ^2^ School of Physical Education, Guizhou Normal University, Guiyang, Guizhou, China; ^3^ Department of Physical Education, Guangzhou Institute of Physical Education, Guangzhou, China; ^4^ College of Physical Education and Health Sciences, Chongqing Normal University, Chongqing, China; ^5^ Department of Orthopaedics, Hainan Hospital of Chinse PLA General Hospital, Sanya, China; ^6^ Nursing Department, The First Medical Center of Chinese PLA General Hospital, Beijing, China; ^7^ Chinese PLA Medical School, Beijing, China

**Keywords:** sleep disturbance, adolescents, machine learning, epidemiology, prediction model, Pittsburgh sleep quality index

## Abstract

**Background:**

Adolescents often experience difficulties with sleep quality. The existing literature on predicting severe sleep disturbance is limited, primarily due to the absence of reliable tools.

**Methods:**

This study analyzed 1966 university students. All participants were classified into a training set and a validation set at the ratio of 8:2 at random. Participants in the training set were utilized to establish models, and the logistic regression (LR) and five machine learning algorithms, including the eXtreme Gradient Boosting Machine (XGBM), Naïve Bayesian (NB), Support Vector Machine (SVM), Decision Tree (DT), CatBoosting Machine (CatBM), were utilized to develop models. Whereas, those in the validation set were used to validate the developed models.

**Results:**

The incidence of severe sleep disturbance was 5.28% (104/1969). Among all developed models, the XGBM model performed best in AUC (0.872 [95%CI: 0.848-0.896]), followed by the CatBM model (0.853 [95% CI: 0.821-0.878]) and DT model (0.843 [95% CI: 0.801-0.870]), whereas the AUC of the logistic regression model was only 0.822 (95% CI: 0.777-0.856). Additionally, the XGBM model had the best accuracy (0.792), precision (0.780), F1 score (0.796), Brier score (0.143), and log loss (0.444).

**Conclusions:**

The XGBM model may be a useful tool to estimate the risk of experiencing severe sleep disturbance among adolescents.

## Introduction

Severe sleep disturbance has emerged as a critical concern for college students, affecting their overall well-being and academic performance (1–4). Recent epidemiological studies indicated that the prevalence of sleep disturbances in this population ranged from 20% to 60% (5), with as many as 12.7% reporting experiencing severe sleep disturbance (6). This widespread issue warrants attention due to its profound implications on both physical and mental health. The consequences of severe sleep disturbances can manifest as excessive daytime sleepiness, reduced cognitive function, and impaired academic performance, which collectively hinder students’ ability to thrive in their educational pursuits (2, 3). Moreover, the long-term repercussions of inadequate sleep can predispose individuals to a spectrum of chronic diseases, including liver disease, diabetes, hypertension, and cardiovascular conditions (7, 8). As the academic pressures and lifestyle changes associated with college life intensify, the need for effective strategies to mitigate sleep disturbances has become increasingly urgent, highlighting the necessity for ongoing research in this critical area.

The causes of sleep disturbance among university students are multifactorial. Academic pressure, social life, and environmental factors are some of the most common causes of sleep disturbance among adolescents. Lifestyle factors such as smoking, alcohol consumption, and caffeine intake can also contribute to sleep disturbance (9). The use of electronic devices, such as smartphones and laptops, is able to disrupt sleep patterns (10). Primary headaches, prevalent among younger populations, can negatively impact sleep quality (11). In addition, there is a significant relationship between sleep disturbance and psychology. Psychological factors such as anxiety, depression, and stress can lead to sleep disturbance (3, 12). Conversely, sleep disturbance can exacerbate psychological problems. Namely, poor sleep quality can increase the risk of developing mood disorders such as depression and anxiety (13).

Notably, a predictive model can help identify adolescents who are at high risk of developing sleep disturbance, and the model can be established based on significant risk factors. By identifying students at high risk of developing sleep disturbance, interventions can be implemented to prevent the development of sleep disturbance. However, the existing literature on predicting severe sleep disturbance is limited, primarily due to the absence of reliable tools. The last decade saw major progress in the field of machine learning with the increase in processing power, and it has emerged as a promising tool for the diagnosis and treatment of sleep problems (14–16). Machine learning algorithms can analyze large amounts of data, identify patterns and relationships, and predict outcomes, making them ideal for analyzing sleep data.

Therefore, this study aims to develop and validate an artificial intelligence (AI) tool to assess the risk of experiencing severe sleep disturbance using machine learning algorithms for adolescents. By identifying individuals who are at risk for developing sleep disorders, healthcare providers can offer early intervention and treatment, reducing the risk of sleep disturbance and thus further improving overall health outcomes. As technology continues to advance, AI prediction tool will become increasingly important approaches in the management of sleep disturbance.

## Methods

### Participants and study design

This study analyzed 1966 university students across five institutions: Xiamen University of Technology (Xiamen), North China University of Water Resources and Electric Power (Zhengzhou), Chongqing Normal University (Chongqing), Harbin Sport University (Harbin), and Sichuan Normal University (Chengdu), during the period from September to December 2021. Participants in this study were volunteers who willingly engaged in the survey, providing responses reflective of their actual circumstances. The survey gathered information regarding their basic characteristics, lifestyle choices, exercise habits, and psychological well-being. Only those participants who consented to participate and were on track for timely graduation were included in the analysis. To ensure accessibility for university students, the survey was conducted online. Of all enrolled participants, 80% were randomly selected as the training set (n=1572), and the remaining 20% were treated as the validation set (n=394). Participants in the training set were used to build the machine learning models. On the other hand, participants in the validation set were used to evaluate and validate the performance of the models. The study protocol was approved by the Academic Committee and Ethics Board of the Xiamen University of Technology (No. 202001), and informed consent was obtained from all subjects or legal guardians before filling the questions in the survey. Participants were all informed that their personal information was not identified and collected, and all data were anonymous. The study was abided by the Declaration of Helsinki.

### Collection of clinical characteristics

This study collected participant’s basic characteristics (age, gender, grade, and marital status), lifestyle (number of cigarettes per day, drinking frequency per week, monthly expense, and prefer eating oily food, barbecue, vegetable, and fruit), sport habit (sedentary time and sport frequency per week), chronic disease, and psychological health (anxiety, depression, and stress), history of sleep disorder, and history of mental distress after thoroughly searching for literature and in terms of availability of variables (3, 12, 17–19). Anxiety was evaluated using the generalized anxiety disorde-7 (GAD-7) (20, 21), and this scale was ranged from 0 to 21 with 0 to 4 indicating none anxiety, 5 to 9 indicating mild anxiety, 10 to 13 indicating moderate anxiety, and 14 and above indicating severe anxiety. In this study, the Cronbach α of GAD-7 was 0.939. Participant’s depression was assessed using the patient health questionnaire-9 (PHQ-9) with the score range of 0 to 27 (20, 21). In this scale, 0 to 4 were categorized as none depression, 5-9 mild depression, 10-14 moderate depression, 15-19 moderate-to-severe depression, and 20-27 severe depression. In the present study, the PHQ-9 had an internal consistency (Cronbach α) of 0.923. Stress was measured using depression anxiety stress scale-21 (DASS-21), and in the subscale to evaluate stress condition, the total score was the two times of the sum of seven questions in the DASS-21 (22). Thus, the stress score ranged from 0 to 41 with a higher score suggesting severer stress. To elaborate, a score of 14 and below indicates normal status, 15-18 indicates mild stress, 19-25 indicates moderate stress, and 26-33 indicates severe stress, and 34-41 indicates extremely severe stress. The stress score had a Cronbach α of 0.950 in the study.

### Definition of serve sleep disturbance

Participant’s sleep quality was evaluated using the Pittsburgh Sleep Quality Index (PSQI) (23). The PSQI is a 19-item scale which is a retrospectively self-report questionnaire to assess seven fields during the past seven days, including sleep latency, habitual sleep efficiency, sleep duration, sleep disturbances, subjective sleep quality, use of sleep medications, and daytime dysfunction. Each item has a score range from 0 representing no difficulty to 3 representing severe difficulty, and after summing the scores of all items, the global score is ranged from 0 to 21. Since the PSQI is a self-report questionnaire, sleep disturbances reflect the subjective experiences of the respondents in our study. Sleep disturbance was defined as the PSQI of above 5 and in the study severe sleep disturbance was defined that participants with the PSQI of above 10 (6, 24–26). The PSQI had an internal consistency (Cronbach α) of 0.746 in the present study, and demonstrated favorable sensitivity.

### Modelling and validation

In the study, we used SMOTETomek, a resampling strategy, to address the effects of imbalanced data distribution to produce a robust model. The SMOTETomek is a combination of Synthetic Minority Oversampling Technique and Tomek Links Undersampling. Participants in the training set were utilized to establish models, and the logistic regression (LR) model and five machine learning algorithms, including the eXtreme Gradient Boosting machine (XGBM), Naïve Bayesian (NB), Support Vector Machine (SVM), Decision Tree (DT), and CatBoosting Machine (CatBM), were utilized to develop machine learning models. Whereas, those in the validation set were used to validate models, and area under the curve (AUC) with applying 100 bootstraps, calibration curve, accuracy, precision, recall, F1 score, Brier score, log loss, and decision curve were severed as evaluation metrics. Machine learning algorithms was applied using Python (version 3.9.7), and hyper parameters tuning tool was based on Python scikit learn (version 1.2.2). The Brier score is a metric used to evaluate the accuracy of probabilistic predictions, particularly in the context of binary outcomes (27). It calculates the mean squared difference between predicted probabilities and actual outcomes, with lower scores indicating better predictive performance. To improve the clinical applicability of predictive models, Shapley additive explanation (SHAP) values are utilized to identify the importance of various features within the model (28–30). SHAP values offer insights into how each feature contributes to the predictions, enabling clinicians to understand which factors are most influential in determining outcomes.

### Establishment of an innovative web-based AI model

The design of the web-based AI model interface was crafted to enable users to efficiently input patient data and obtain accurate predicted probabilities. It features user-friendly panels that facilitate the selection of model parameters, probability calculations, and easy access to comprehensive information about the underlying model. The primary objective of the interface is to provide a captivating and interactive experience, enhancing users’ ability to interpret and evaluate the likelihood of severe sleep disturbances.

### Comparative analysis: assessing the predictive performance of the AI tool vis-à-vis medical experts

To ascertain the efficacy of the AI tool, a comparative study was conducted pitting the tool against seasoned psychologists with extensive experience in the field. Five esteemed psychologists participated in this study independently, offering their individual predictions concerning the risk of severe sleep disturbance. This study provided 100 participants, whom the psychologists assessed based on their experience to evaluate the risk of severe sleep disturbance. Subsequently, by comparing the predicted outcomes with the actual status of sleep disturbance, the AUC value was used as an evaluation metric for the predictive performance of each expert.

### Statistical analysis

This study summarized the continuous variables as the format of average and standard deviation (SD); the categorical variables were presented as the format of proportions. Chi-square test was used to compare the distribution of categorical variables, and student *t* test or Wilcoxon rank test was used to make the comparison of continuous variables. All statistical analysis was conducted using R language program (version 4.1.2). A P value of less than 0.05 was considered as significant with two sides.

## Results

### Participant’s basic characteristics

A total of 1969 participants with an average age of 19.65 years (SD: 1.71) were enrolled for analysis (**Table 1**). Of all enrolled participants, 55.2% were female, 47.2% were second grade, and 76.8% were single. The majority of participants were non-current smoker (95.2%), non-current drinker (86.4%), and had a monthly expense of less than 2000 yuan (79.1%). Regarding eating habit, the proportion of preference to eat oily food, barbecue, vegetable, and fruit were 26.0%, 28.9%, 49.3%, and 57.6%, respectively. Regarding sport, 78.7% participants did sport once a week or above, and 76.5% participants had sedentary time of three hours or above a day. In addition, only 4.1% of participants had chronic disease.

**Table 1:** Participant’s characteristics and a comparison stratified by the presence of severe sleep disturbance among university students.

Characteristics	Overall	Severe sleep disturbance		P
		No	Yes	
n	1966	1862	104	
Age (years, mean [SD])	19.65 (1.71)	19.64 (1.71)	19.79 (1.75)	0.401
Gender (Male/Female, %)	880/1086 (44.8/55.2)	836/1026 (44.9/55.1)	44/60 (42.3/57.7)	0.678
Grade (%)				0.157
First	482 (24.5)	465 (25.0)	17 (16.3)	
Second	927 (47.2)	868 (46.6)	59 (56.7)	
Third	335 (17.0)	318 (17.1)	17 (16.3)	
Fourth	222 (11.3)	211 (11.3)	11 (10.6)	
Marital status (%)				0.717
Single	1510 (76.8)	1428 (76.7)	82 (78.8)	
Dating	445 (22.6)	424 (22.8)	21 (20.2)	
Married	11 (0.6)	10 (0.5)	1 (1.0)	
Number of cigarettes per day (%)				0.315
0	1871 (95.2)	1775 (95.3)	96 (92.3)	
1-3	30 (1.5)	29 (1.6)	1 (1.0)	
4-6	34 (1.7)	30 (1.6)	4 (3.8)	
7-9	7 (0.4)	6 (0.3)	1 (1.0)	
≧10	24 (1.2)	22 (1.2)	2 (1.9)	
Drinking frequency per week (%)				0.006
0	1698 (86.4)	1613 (86.6)	85 (81.7)	
1	222 (11.3)	209 (11.2)	13 (12.5)	
2-3	34 (1.7)	31 (1.7)	3 (2.9)	
4-5	7 (0.4)	6 (0.3)	1 (1.0)	
≧6	5 (0.3)	3 (0.2)	2 (1.9)	
Monthly expense (¥, %)				<0.001
0-1999	1556 (79.1)	1474 (79.2)	82 (78.8)	
2000-4999	394 (20.0)	376 (20.2)	18 (17.3)	
5000-9999	8 (0.4)	8 (0.4)	0 (0.0)	
≧10000	8 (0.4)	4 (0.2)	4 (3.8)	
Prefer eating oil food (No/Yes, %)	1454/512 (74.0/26.0)	1385/477 (74.4/25.6)	69/35 (66.3/33.7)	0.089
Prefer eating barbecue (No/Yes, %)	1397/569 (71.1/28.9)	1336/526 (71.8/28.2)	61/43 (58.7/41.3)	0.006
Prefer eating vegetable (No/Yes, %)	997/969 (50.7/49.3)	936/926 (50.3/49.7)	61/43 (58.7/41.3)	0.118
Prefer eating fruit (No/Yes, %)	833/1133 (42.4/57.6)	783/1079 (42.1/57.9)	50/54 (48.1/51.9)	0.268
Sedentary time (hours, %)				0.628
<1	100 (5.1)	94 (5.0)	6 (5.8)	
≧1 and <3	362 (18.4)	347 (18.6)	15 (14.4)	
≧3 and <6	655 (33.3)	622 (33.4)	33 (31.7)	
≧6	849 (43.2)	799 (42.9)	50 (48.1)	
Sport frequency per week (%)				0.055
0	418 (21.3)	385 (20.7)	33 (31.7)	
1-2	715 (36.4)	685 (36.8)	30 (28.8)	
3-4	415 (21.1)	395 (21.2)	20 (19.2)	
≧5	418 (21.3)	397 (21.3)	21 (20.2)	
Chronic disease (No/Yes, %)	1885/81 (95.9/4.1)	1792/70 (96.2/3.8)	93/11 (89.4/10.6)	0.002
GAD-7 (mean [SD])	3.26 (4.03)	2.98 (3.69)	8.24 (6.04)	<0.001
Severity of anxiety (%)				<0.001
None	1350 (68.7)	1321 (70.9)	29 (27.9)	
Mild	481 (24.5)	439 (23.6)	42 (40.4)	
Moderate	95 (4.8)	81 (4.4)	14 (13.5)	
Severe	40 (2.0)	21 (1.1)	19 (18.3)	
PHQ-9 (mean [SD])	4.17 (4.90)	3.78 (4.47)	11.17 (6.71)	<0.001
Severity of depression				<0.001
None	1222 (62.2)	1207 (64.8)	15 (14.4)	
Mild	526 (26.8)	492 (26.4)	34 (32.7)	
Moderate	136 (6.9)	109 (5.9)	27 (26.0)	
Moderate-to-severe	55 (2.8)	40 (2.1)	15 (14.4)	
Severe	27 (1.4)	14 (0.8)	13 (12.5)	
Stress score (mean [SD])	7.99 (8.27)	7.41 (7.78)	18.29 (9.97)	<0.001
Severity of stress (%)				<0.001
None	1619 (82.3)	1578 (84.7)	41 (39.4)	
Mild	134 (6.8)	120 (6.4)	14 (13.5)	
Moderate	132 (6.7)	106 (5.7)	26 (25.0)	
Severe	60 (3.1)	44 (2.4)	16 (15.4)	
Extremely severe	21 (1.1)	14 (0.8)	7 (6.7)	
History of sleep disorder (No/Yes, %)	1912/54 (97.3/2.7)	1827/35 (98.1/1.9)	85/19 (81.7/18.3)	<0.001
History of mental distress (No/Yes, %)	1920/46 (97.7/2.3)	1830/32 (98.3/1.7)	90/14 (86.5/13.5)	<0.001
PSQI (mean [SD])	5.54 (2.93)	5.16 (2.48)	12.35 (1.74)	<0.001
Sleep latency (mean [SD])	1.12 (0.96)	1.04 (0.91)	2.56 (0.64)	<0.001
Habitual sleep efficiency (mean [SD])	0.33 (0.70)	0.26 (0.60)	1.48 (1.12)	<0.001
Sleep duration (mean [SD])	0.92 (0.79)	0.86 (0.76)	1.88 (0.62)	<0.001
Sleep disturbances (mean [SD])	0.80 (0.59)	0.77 (0.57)	1.49 (0.64)	<0.001
Subjective sleep quality (mean [SD])	0.98 (0.70)	0.93 (0.65)	2.02 (0.70)	<0.001
Use of sleep medications (mean [SD])	0.05 (0.31)	0.02 (0.19)	0.49 (1.01)	<0.001
Daytime dysfunction (mean [SD])	1.34 (0.95)	1.28 (0.92)	2.43 (0.65)	<0.001

SD, Standard deviation; GAD-7, Generalized anxiety disorde-7; PHQ-9, Patient health questionnaire; PSQI, Pittsburgh Sleep Quality Index.

### Mental health and quality of sleep

In the entire population, the average of GAD-7 was 3.26, and 31.3% participants had mild or above anxiety. As for depression, the average PHQ-9 was 4.17, and 37.8% participants had mild or above depression. The means stress score was 7.99, and 17.7% participants had mild or above stress. The history of sleep disorder and mental distress is not common, because the two accounted for 2.7% and 2.3%, respectively. The average PSQI was 5.54, and 5.28% (104/1969) participants had severe sleep disturbance.

### Analysis of participants categorized by the presence of severe sleep disturbance

Individuals experiencing severe sleep disturbances were found to exhibit a higher frequency of alcohol consumption per week (P=0.006) (**Table 1**), increased monthly expenditures (P<0.001), a preference for consuming barbecued food (P=0.006), the presence of chronic illnesses (P=0.002), heightened levels of anxiety (P<0.001), depression (P<0.001), and stress (P<0.001), as well as a history of sleep disorders (P<0.001) or psychological distress (P<0.001). Thus, the above variables were used as input features for modelling.

### Evaluation of models

**Figure 1:**
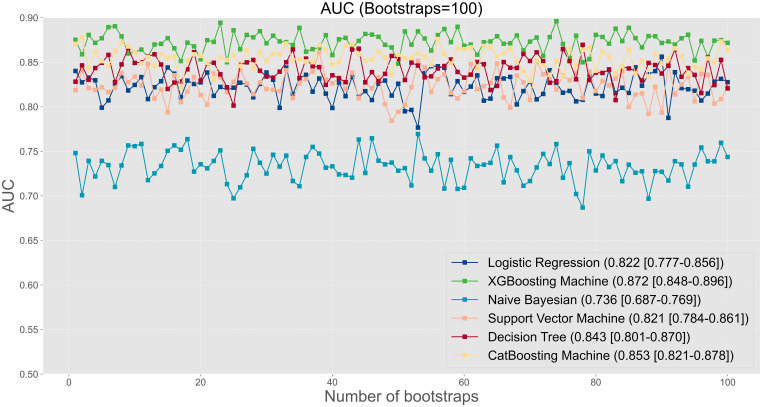
The area under the curve for all developed models after applying 100 bootstraps.

**Figure 2:**
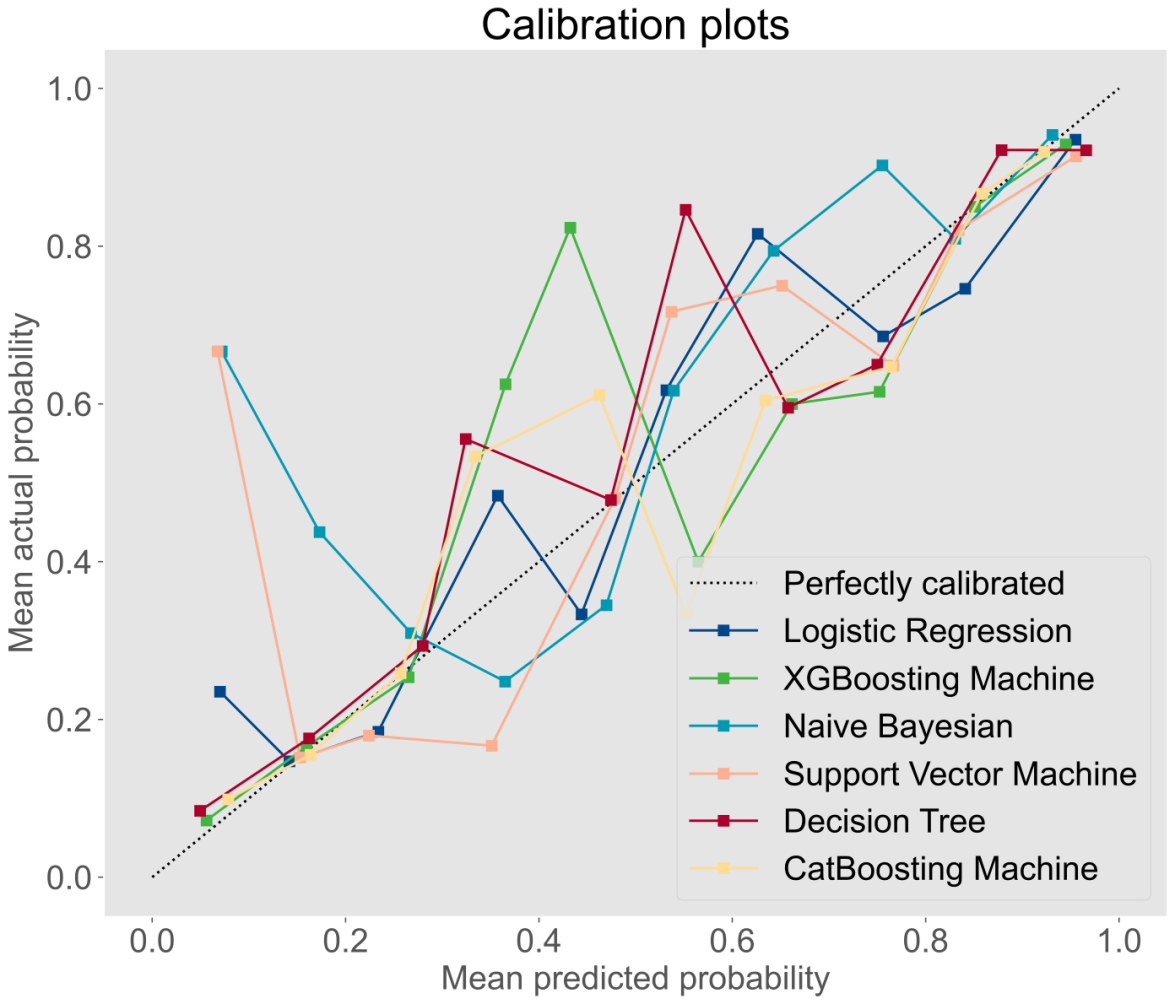
Calibration curve for all developed models.

**Figure 3:**
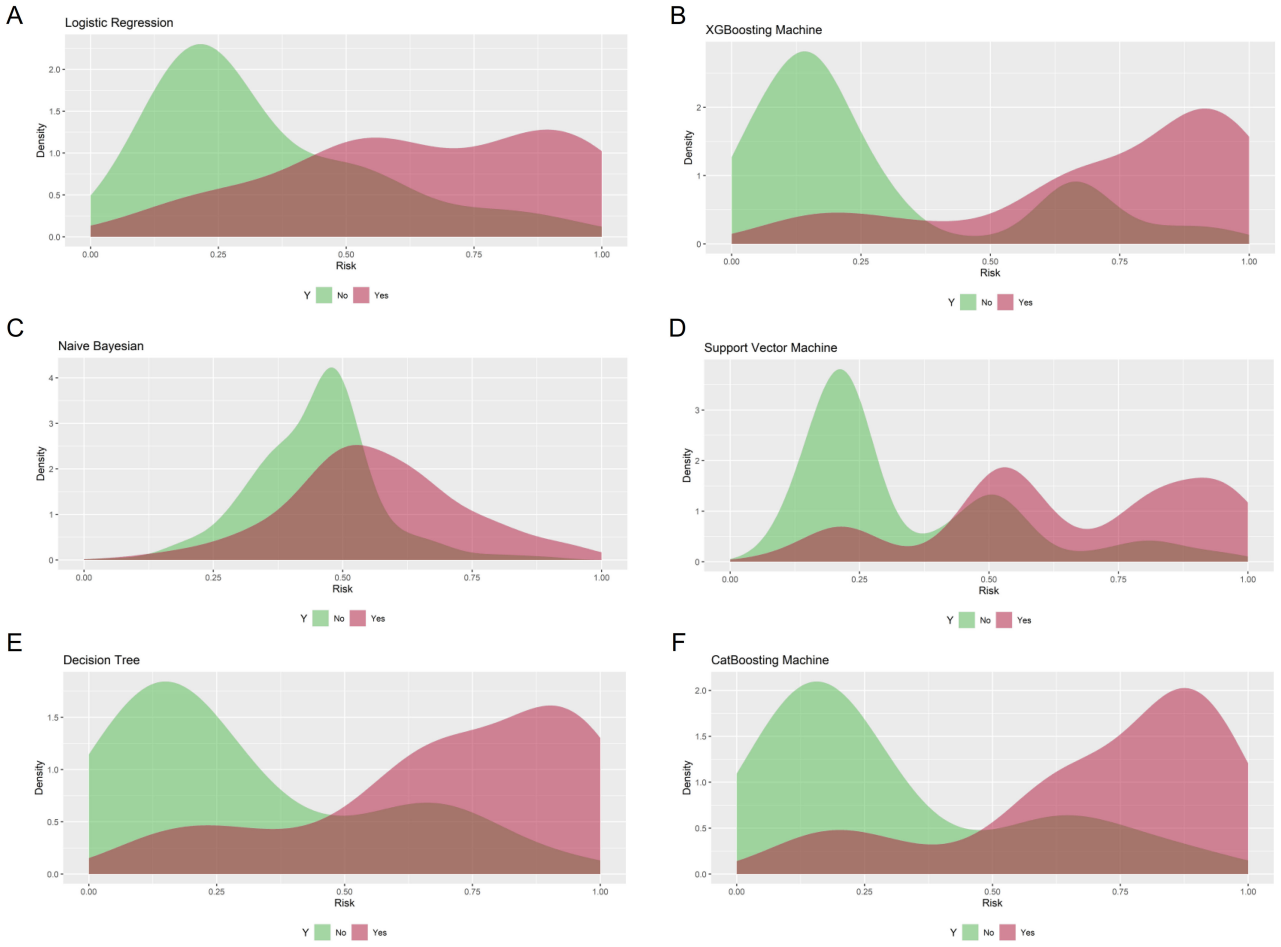
Density curves for all developed models. **(A)** Logistic regression; **(B)** XGBoosting Machine; **(C)** Naïve Bayesian; **(D)** Support Vector Machine; **(E)** Decision Tree; **(F)** CatBoosting Machine. The green curve indicates patients without severe sleep disturbance, and the red curve indicates patients with severe sleep disturbance.

The prediction performance was evaluated using AUC, calibration curve, accuracy, precision, recall, F1 score, Brier score, and log loss. **Figure 1** shows AUC of all models after applying 100 bootstraps, and it demonstrated that the XGBM model performed best in AUC (0.872 [95%CI: 0.848-0.896]), followed by the CatBM model (0.853 [95% CI: 0.821-0.878]) and DT model (0.843 [95% CI: 0.801-0.870]). However, the NB model was the worst in AUC (0.736 [95% CI: 0.687-0.769]), and the LR model was the second worst in AUC (0.822 [95% CI:0.777-0.856]). **Figure 2** shows the calibration curves of all models, and it proved that the most of models, in particular the XGBM model, had favorable calibrating ability. Less overlap between participants with and without severe sleep disturbance was observed, especially in the XGBM, DT, and CatBM models (**Figure 3**). This result denoted that good separation of predicted risk was achieved in the three models between participants who had severe sleep disturbance and who had not. By contract, relatively large overlap existed in the LR, NB, and SVM models.

**Figure 4:**
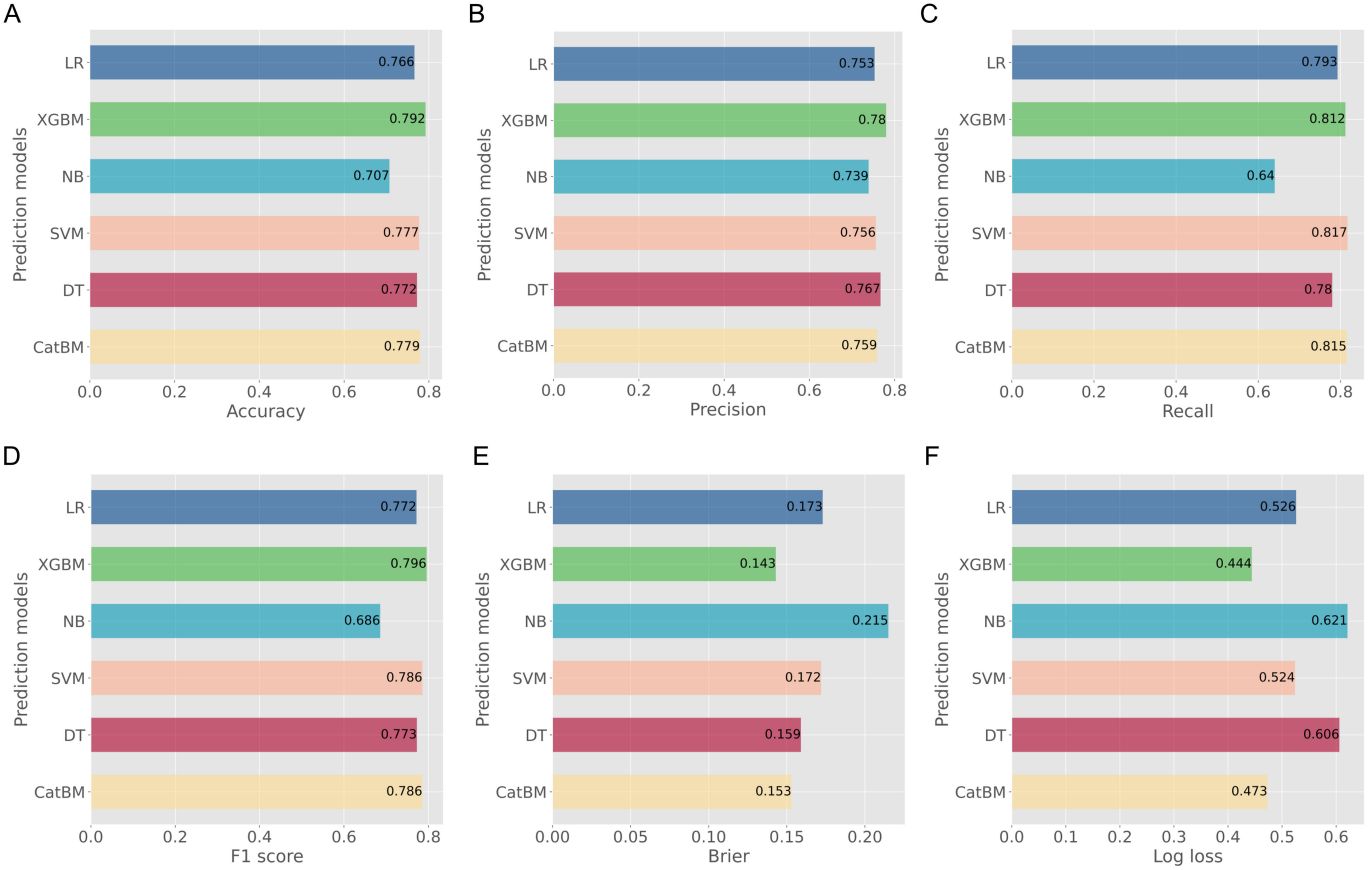
Evaluation metrics of prediction performance for all developed models. **(A)** Accuracy; **(B)** Precision; **(C)** Recall; **(D)** F1 score; **(E)** Brier; **(F)** Log loss.

**Figure 5:**
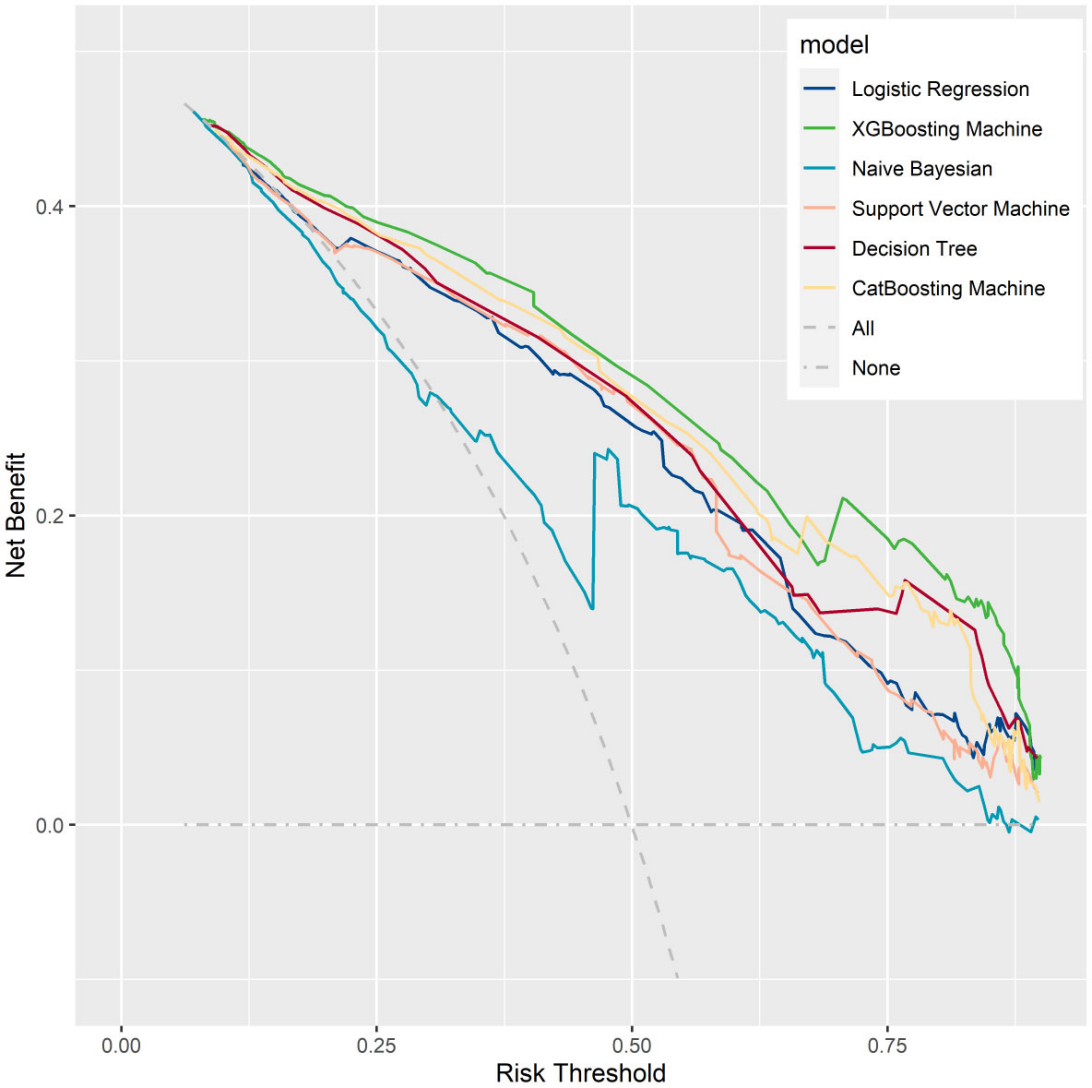
Decision curve analysis for all developed models. It examined the net benefit of using a model for predicting outcomes across a range of threshold probabilities.

More metrics are summarized in **Table 2** and **Figure 4**. The XGBM model also had the best accuracy (0.792), precision (0.780), F1 score (0.796), Brier score (0.143), and log loss (0.444). Although the CatBM performed the best in recall (0.815), the recall of XGBM was also good (0.812). Thus, the XGBM was the optimal model based on the above findings. In addition, to assess the goodness of different models, we first calculated the net benefit for each model at different threshold probabilities. The net benefit considered the consequences of true positives (benefit) and false positives (harm) in making predictions. Next, we plotted the decision curves for each model, with the x-axis representing the threshold probability and the y-axis representing the net benefit. We were able to compare the decision curves of different models to assess their relative performance, when putting the decision curves of all models together. The XGBM model had the best clinical net benefits (**Figure 5**), as its decision curve had higher net benefit over a wide range of threshold probabilities, indicating that the model provided more accurate predictions, which was able to minimize false positives while maximize true positives.

**Table 2:** Prediction performance of models to estimate the risk of severe sleep disturbance among university students.

Metrics	Models					
	Logistic Regression	XGBoosting Machine	Naïve Bayesian	Support Vector Machine	Decision Tree	CatBoosting Machine
AUC (95% CI)	0.822[0.777-0.856]	0.872[0.848-0.896]	0.736[0.687-0.769]	0.821[0.784-0.861]	0.843[0.801-0.870]	0.853[0.821-0.878]
Accuracy	0.766	0.792	0.707	0.777	0.772	0.779
Precision	0.753	0.780	0.739	0.756	0.767	0.759
Recall	0.793	0.812	0.640	0.817	0.780	0.815
F1 score	0.772	0.796	0.686	0.786	0.773	0.786
Brier	0.173	0.143	0.215	0.172	0.159	0.153
Log loss	0.526	0.444	0.621	0.524	0.606	0.473

AUC, Area under the curve; CI, Confident interval.

### Feature importance

**Figure 6:**
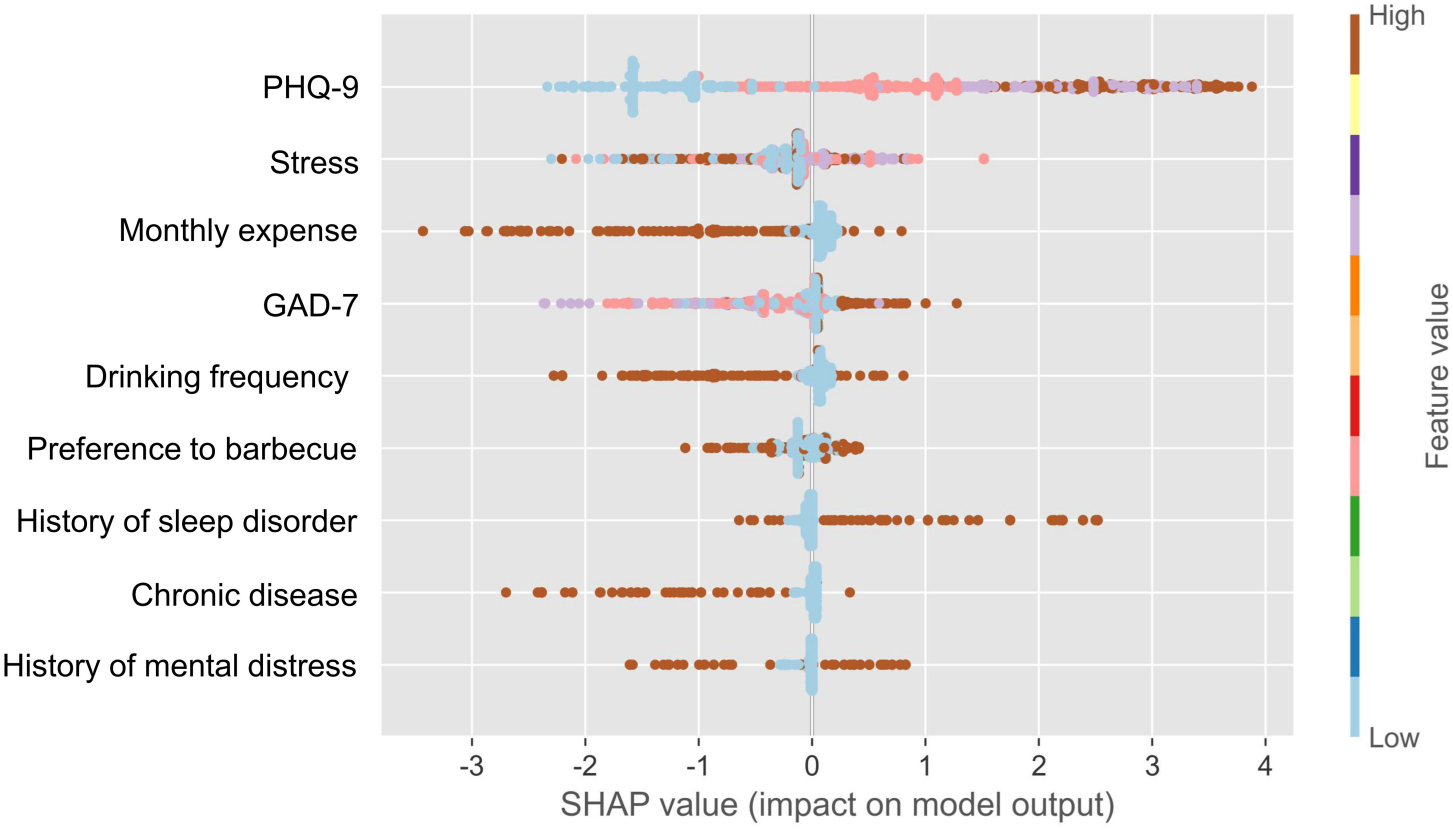
Feature importance using the SHAP analysis.

**Figure 7:**
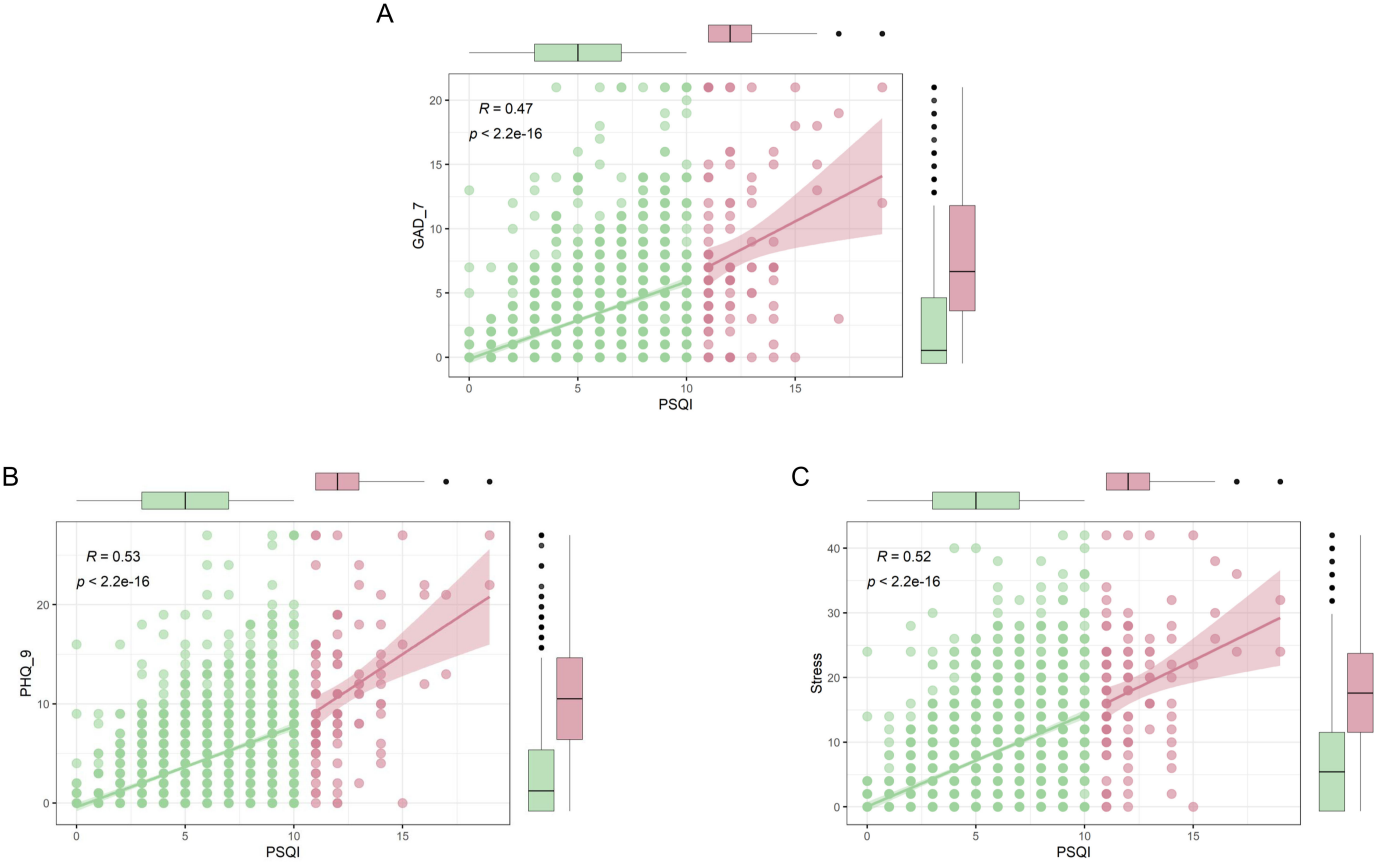
Association between sleep quality and mental health. **(A)** Relationship between PSQI and GAD-7; **(B)** Relationship between PSQI and PHQ-9; **(C)** Relationship between PSQI and stress. Red indicates participants with severe sleep disturbance; Green indicates participants without severe sleep disturbance. GAD-7, Generalized anxiety disorde-7; PHQ-9, Patient health questionnaire; PSQI, Pittsburgh Sleep Quality Index.

**Figure 8:**
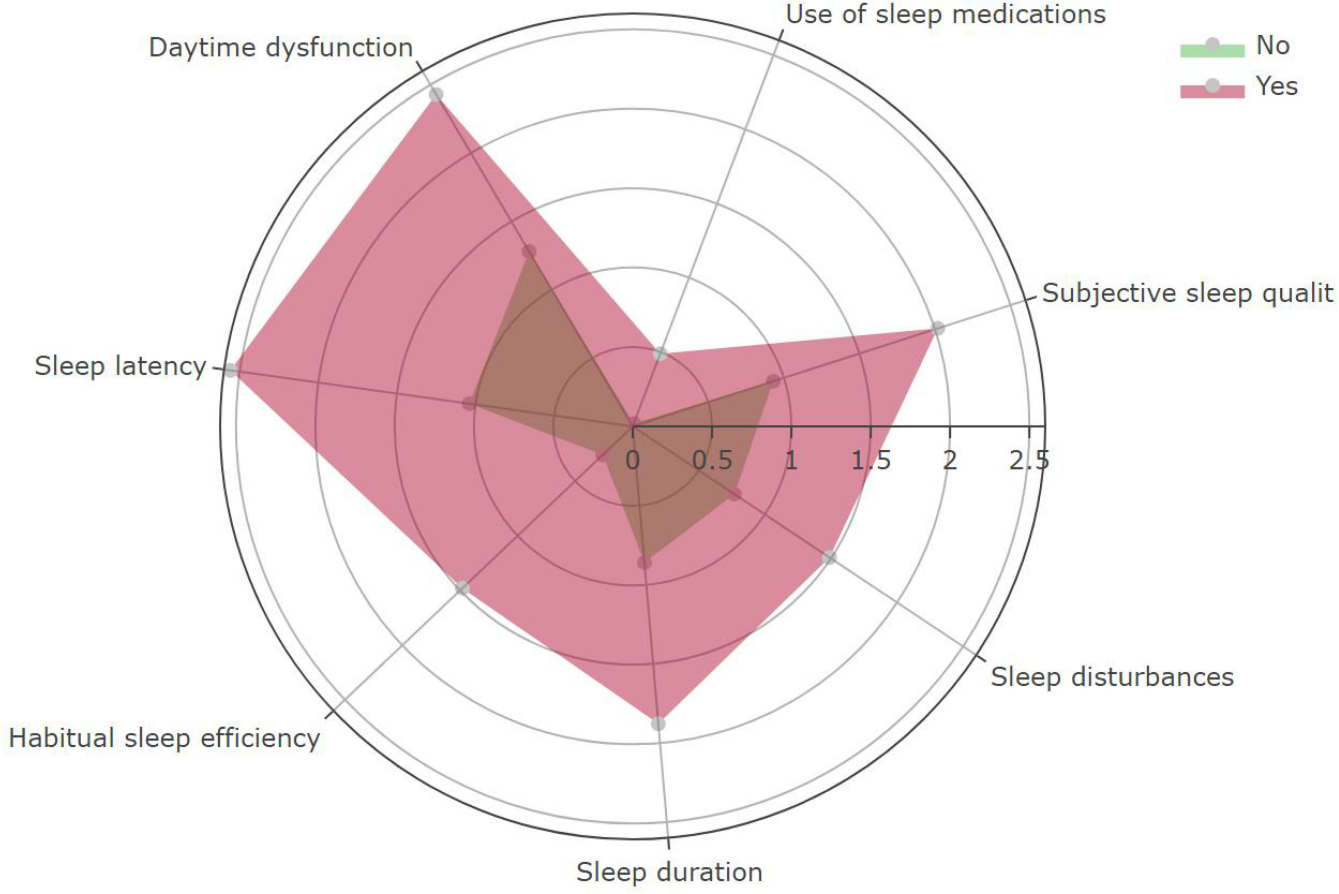
The distribution of the seven subscales of the PSQI between participants with and without severe sleep disturbance. Red indicates participants with severe sleep disturbance; Green indicates participants without severe sleep disturbance.

SHAP-based feature importance analysis showed that the top four important model features in the XGBM model were depression, stress, monthly expense, and anxiety (**Figure 6**). This finding indicated that participant’s mental health was closely related to their quality of sleep. **Figure 7** confirms that the PSQI was significantly associated with GAD-7 (P<0.001), PHQ-9 (P<0.001), and stress score (P<0.001). In addition, participants with the severe sleep disturbance had significantly higher GAD-7 (P<0.001), PHQ-9 (P<0.001), and stress score (P<0.001) than patients without the severe sleep disturbance. Participants with severe sleep disturbance had significantly higher PSQI than patients without severe sleep disturbance (12.35 vs. 5.16). Similar tendency was also observed in the seven subscales of the PSQI (**Figure 8**).

### Applicability of the AI tool

**Figure 9:**
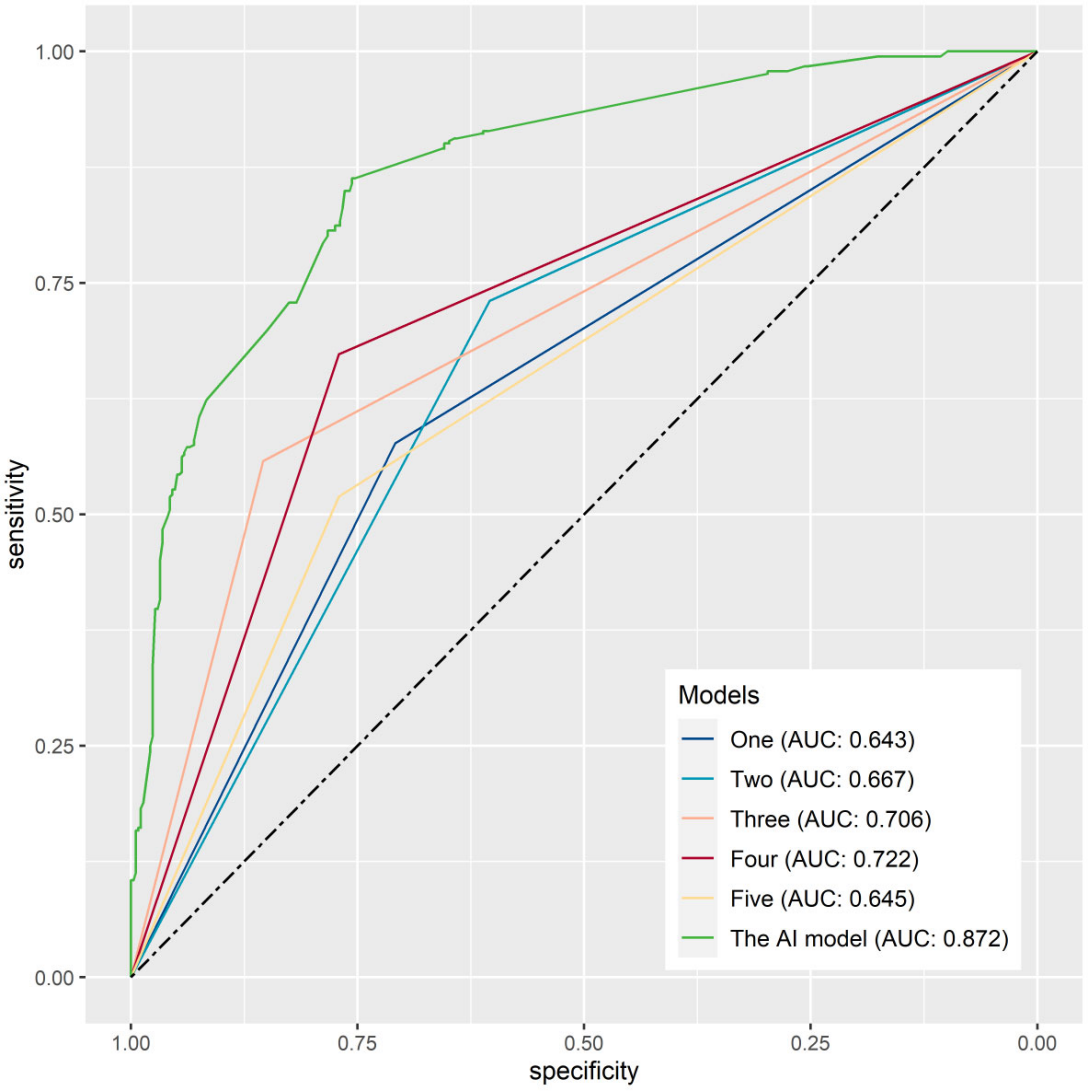
The area under the curve for a comparison of prediction performance between human and the AI tool.

We have developed a user-friendly, web-based AI tool designed to evaluate the risk of severe sleep disturbance, and the code can be available at https://github.com/Starxueshu/severe_sleep_disturbance. For example, a university student does not consume alcohol, has a monthly expense ranging from 0 to 1999 RMB, does not enjoy eating barbecue, has no chronic diseases, experiences moderate anxiety and moderate-to-severe depression, endures severe stress, and has no history of sleep disorders or mental distress. By inputting the above data into the AI tool, we can determine that the predicted risk for this individual to suffer from severe sleep disturbance is 86.95%. In addition, the predictive accuracy of the five psychologists was notably subpar, with AUC3 values ranging between 0.643 and 0.722. This performance was significantly inferior compared to the AI tool’s AUC value of 0.872 (**Figure 9**), highlighting the AI tool’s superior predictive capabilities.

## Discussion

### Principal findings

The study established and validated an AI tool to predict the risk of severe sleep disturbance among adolescents. The AI tool was developed based on the XGBM model, which performed the best with the AUC of 0.872. This tool considered various psychological and lifestyle factors, enabling early identification and intervention for sleep-related issues in this population. This tool can be used by healthcare professionals, parents, and educators to identify individuals at risk and provide timely interventions.

### Risk factors related to sleep disturbance

In the present study, we found that the risk factors that were relevant to severe sleep disturbance included drinking, monthly expense, barbecue, chronic disease, anxiety, depression, stress, and history of sleep disorder and mental distress. In addition, preferring to eating oily food tended to be a risk factor, and loving sport tended to be a protective factor, but the two factors were not significant. Previous studies have shown that alcohol consumption was a significant contributor to poor quality of sleep (17, 18), and this was consistent with our findings. Additionally, individuals who tended to feel more alert upon waking tended to consume more alcohol on average (17). Individuals who spend more money each month were more likely to experience poor sleep quality and insomnia. This may be due to financial stress and worry, as well as the lifestyle factors that often accompany high levels of spending. One study found that individuals who reported high levels of debt and financial strain were more likely to experience insomnia and other sleep disorders (19). Research has shown that sleep disorders were associated with an increased risk of developing a variety of chronic diseases, such as diabetes, hypertension, and cardiovascular disease. One of the main ways in which sleep disorders contribute to chronic diseases is through their effect on the body’s hormonal, metabolic systems, and inflammation (31, 32).

In the present study, the SHAP analysis demonstrated that the top four important model features were depression, stress, monthly expense, and anxiety, indicating the importance of mental health in impacting quality of sleep among university students. Mental health could have a significant impact on the sleep disturbance among college students. Conversely, sleep disturbance was able to intrigue and exacerbate psychological problems (13). Studies have shown that poor global sleep quality could be functioned as a mediator of the prospective bidirectional anxiety-depression relationship (33). Some studies also pointed out that gender, age, and smoking might be risk factors, and eating fruit could be a protective factor (34).

### Applications of AI in sleep disorders

AI has been used in the field of sleep disorders (35). For example, machine learning can be used to analyze sleep data and identify patterns that can be used to diagnose and treat insomnia. In 2021, Kusmakar et al. (36) proposed a novel AI model based on the actigraphy signals to assess chronic insomnia after analyzing 40 cohabiting couples with one partner seeking treatment for insomnia. More recently, Japanese researchers utilized machine learning algorithms to establish models to predict comorbid insomnia among breast cancer patients using a nationwide questionnaire survey, and the AUC of the optimal model was 0.76 (37). Additionally, machine learning was used to diagnose sleep apnea after analyzing sleep data, such as electrocardiographic, oximetric, and polysomnographic recordings. For instance, Simegn et al. (38) used machine learning algorithms to develop an automatic sleep apnea, a potentially serious sleep disorder and characterized by breathing pauses during sleep, and evaluate the severity classification based on the electrocardiograph recordings and saturation of oxygen signals, and the AUC of the model was not assessed in the original study. Zhuang et al. (39) developed a detection framework to assess sleep apnea with the polysomnography data from the radar system after using random forest machine learning, and the accuracy of the detection framework was up to more than 95%. In addition, after summarizing 63 studies undergoing diagnostic model development, a systematic review found that the best AUC was 0.98 in a logistic model with age, waist circumference, Epworth Sleepiness Scale score, and oxygen saturation being model features (40). Interestingly, Kim et al. (41) used a wearable digital device to collect circadian rhythm-based features and proposed a machine learning-based prediction model for assessing attention-deficit and sleep problems among children, and the AUC of model to predict sleep problems was 0.737.

However, the majority of the above machine learning models were developed using the data from actigraphic signals, electrocardiograph recordings, saturation of oxygen signals, and polysomnography, wearable digital devices, making those models hard to be widely used among general populations to screen patients at the high risk of experiencing severe sleep disorders. Furthermore, those models were not developed especially for adolescents, and thus their effectiveness might be limited in this population. Notably, a study developed a prediction model after investigating potential correlated factors, and the model was established using machine learning techniques with age, number of cups of tea, electronics usage hours, headache, other systematic diseases, and neck pain being model features. But the optimal machine learning model was random forest model with an AUC of only 0.74 (42). In addition, we previously developed a machine learning-based model to assess sleep disturbance among college students, achieving an optimal AUC value of 0.779 (43). In the present study, we specifically focused on severe sleep disturbance, and our results demonstrate improved predictive performance, with the new model attaining a maximum AUC value of 0.872, indicating enhanced predictive power. Overall, this research presents a more effective model for identifying severe sleep disturbance, thereby increasing its clinical relevance.

With AI assistance, the risk of sleep disturbances among university students has been identified. Based on previous literature, those at high risk of sleep disorders should establish a regular sleep routine, avoiding daytime naps, and limiting caffeine and alcohol, especially in the evening. Creating a quiet, dark, and cool sleep environment while minimizing electronic distractions is also crucial. Relaxation techniques like deep breathing or meditation may help those who struggle to fall asleep. Low-risk individuals should maintain good sleep habits, avoid late-night screen time, reduce stress, and engage in regular physical activity. They can optimize their sleep environment with a comfortable mattress, pillows, and by using white noise or blackout curtains. In both cases, consulting a healthcare provider or sleep specialist may be necessary if sleep problems persist despite these measures.

In addition, our study demonstrated that abstaining from drinking, less monthly expense, avoiding barbecue and oily food, treating chronic disease, loving sports, and alleviating anxiety, depression, and stress are able to do some help to improve quality of sleep. Therefore, interventions such as counseling, lifestyle changes, and behavioral therapies can be implemented to prevent the development of sleep disturbance. Furthermore, based on previous studies, interventions for sleep disorders in college students, such as cognitive-behavioral therapy and sleep hygiene education, have shown effectiveness in improving sleep quality (44, 45). However, evidence on the effectiveness of sleep education remains insufficient (46). Additional strategies include relaxation techniques, physical activity, and exposure to natural light (47). Overall, these interventions and preventive measures can be effective in addressing sleep disorders in college students and improving overall well-being.

### Limitations

Several limitations still exist in the study. To begin with, the data collected for the study might be biased due to self-reporting, and thus recall bias was hardly be avoided and it might affect the accuracy of the results. Secondly, although this study collected extensive features in terms of demographics, sports, lifestyles, and mental conditions, it might not capture all relevant variables that contribute to sleep disorders among college students. Thirdly, the study might not be able to establish a causal relationship between the identified predictors and sleep disorders among college students, due to confounding variables or the design of the study. Fourthly, polysomnography remains the sole objective method for evaluating sleep disorders; in contrast, other validated questionnaires, such as PSQI used in the study, often exhibit various limitations, including a notable degree of subjectivity. Lastly, the model used in the study might not be generalizable to other populations or contexts, which may limit the utility of the findings. Therefore, although this study had a relatively large size of sample and the model had favorable prediction performance, this study still needs wide external validation.

## Conclusions

The XGBM model can be a favorable method to estimate the risk of severe sleep disturbance among university students, and the online calculator is able to be used as a screen tool to individually identify those who are at high risk of sleep disturbance. Thus, earlier intervention and improved outcomes can be achieved. This study also demonstrates that Participants with severe sleep disturbance usually suffer from poor psychological health, and abstaining from drinking, less monthly expense, avoiding barbecue, treating chronic disease, and alleviating anxiety, depression, and stress are able to do some help to improve quality of sleep among university students.

## Data Availability

The raw data supporting the conclusions of this article will be made available by the authors, without undue reservation.
